# Biobased Lignin as a Sustainable Flame Retardant Additive for Recycled Polyester Fabrics

**DOI:** 10.1021/acsomega.6c00738

**Published:** 2026-05-29

**Authors:** Dujdow Niyomdacha, Thanika Hutakamol, Chutima Vanichvattanadecha, Penwisa Pisitsak

**Affiliations:** a Department of Materials and Textile Technology, Faculty of Science and Technology, 65100 Thammasat University, Pathum Thani 12121, Thailand; b Advanced Composite and Nanotextiles Research Team, National Nanotechnology Center, National Science and Technology Development Agency, Pathum Thani 12120, Thailand; c Center of Excellence on Petrochemical and Materials Technology, Chulalongkorn University, Bangkok 10330, Thailand

## Abstract

Recycled poly­(ethylene terephthalate) (rPET) textiles present intrinsic challenges for flame-retardant finishing due to the hydrophobic and chemically inert surface of PET fibers, as well as their pronounced melt-dripping behavior during combustion. Here, we demonstrate that incorporating only 1% owf lignin into a phosphorus–nitrogen coating is sufficient to achieve effective flame retardancy (LOI of 37.5%, UL 94 V-0), while retaining self-extinguishing behavior after five laundering cycles. A sustainable halogen-free coating system was developed by combining a phosphorus-based flame retardant (aluminum diethylphosphinate) with biobased lignin and sericin within a cross-linked poly­(vinyl alcohol)/citric acid matrix. The incorporation of lignin promoted char formation, suppressed melt dripping, and facilitated rapid self-extinguishing behavior. Increasing lignin loading to 3–5% owf did not further improve UL 94 performance and instead reduced wash durability, indicating that low lignin loading is sufficient for effective performance. In addition to enhanced flame retardancy, the treated fabrics maintained mechanical integrity, exhibited hydrophobic surface characteristics, and showed good colorfastness to crocking.

## Graphical abstract

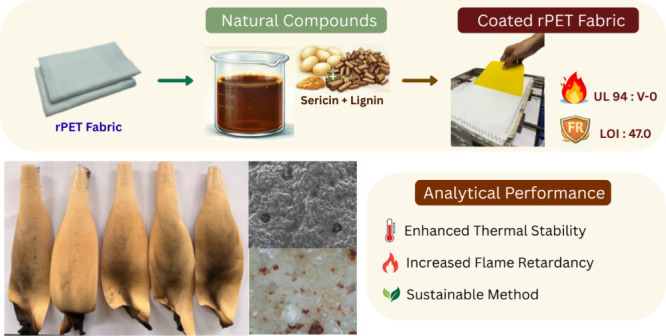

## 1 Introduction

Despite extensive research on lignin-based flame-retardant systems in natural fibers and their composites,^1^ their application to polyester textiles remains challenging. Cellulosic fibers contain abundant hydroxyl groups that enable interactions and chemical modification with lignin,^2^ whereas poly­(ethylene terephthalate) (PET) fibers possess a hydrophobic and chemically inert surface with limited reactive functional groups.^3^ In addition, PET textiles, which represent the most widely used synthetic fibers in the global textile industry, exhibit pronounced melt-dripping behavior during combustion.^4^ This phenomenon can accelerate flame propagation and increase fire hazards. Flame-retardant treatments for PET textiles can be introduced either during polymer melt spinning or through postfinishing on fabrics.^5^ While melt incorporation can provide durable flame retardancy, it requires modification at the fiber production stage. In contrast, postfinishing treatments applied to fabric substrates are more flexible and widely used in the textile industry, but achieving durable adhesion on chemically inert PET surfaces remains challenging.^6^

Notably, PET is a recyclable polymer with recycling rates approaching 100%, and recycled PET (rPET) has been widely utilized in the textile sector, particularly for staple fiber production, followed by its use in food and beverage packaging.^7^ Conventional flame-retardant (FR) treatments for PET and rPET have largely relied on halogenated additives, whose use has increasingly been restricted due to concerns regarding toxicity and environmental persistence. Phosphorus-based flame retardants (P-FRs) have emerged as promising alternatives because they enhance polymer flame retardancy primarily through the promotion of char formation. This mechanism reduces heat release, suppresses melt dripping and flame propagation, and avoids the release of toxic byproducts commonly associated with halogenated systems. P-FRs are widely employed in intumescent flame-retardant systems, which typically comprise an acid source, a carbon source, and a gas source. Upon heating, the P-FR decomposes to generate phosphoric or polyphosphoric acid species that catalyze the formation of carbonaceous char. Simultaneously, the gas source releases nonflammable gases that expand the char structure, forming a multicellular barrier that limits heat and mass transfer between the flame and the substrate.^8,9^ Furthermore, the presence of nitrogen-containing compounds is known to enhance flame retardancy through phosphorus–nitrogen (P–N) synergistic effects, which promote char formation and the release of inert gases during combustion.^9^

Lignin is a polyphenolic biopolymer that constitutes a major component of plant cell walls^10^ and has attracted increasing attention as a sustainable flame-retardant additive. Due to its inherently cross-linked three-dimensional aromatic structure and high carbon content, lignin undergoes dehydration, condensation, and aromatization upon heating, leading to the formation of a thermally stable carbonaceous char. This char layer suppresses heat and oxygen transfer while limiting the release of flammable volatiles, thereby protecting the underlying material from further combustion. Lignin has been reported to thermally degrade above 150 °C and to yield a stable carbonaceous char at temperatures up to approximately 700 °C.^11^

Recent studies have demonstrated the effectiveness of lignin-based flame-retardant systems in textile applications; however, most of these studies have been conducted on cellulose-based substrates. For instance, cotton fabrics treated with a multifunctional layer-by-layer nanocoating based on a lignin derivative exhibited self-extinguishing behavior even with a low number of deposited layers.^12^ Similarly, the incorporation of lignin-based flame retardants significantly reduced burning time and flame height in cotton fabrics.^13^ More recently, the combination of alkali lignin and ammonium polyphosphate enhanced char formation and enabled treated cotton fabrics to achieve a V-0 rating in the UL 94 test (Underwriters Laboratories Standard 94), with the limiting oxygen index (LOI) increasing from 18.6% to 48.5%.^14^

Despite these promising results, studies investigating lignin-based treatments for PET fibers or PET-based textiles have mainly focused on improving mechanical properties,^15–18^ antimicrobial activity,^19,20^ or ultraviolet protection^15,21^ rather than flame retardancy. To date, only a limited number of studies have reported the use of lignin for enhancing the flame retardancy of PET fabrics^22–24^ and among these, only one study evaluated wash durability,^22^ which was limited to two dry cleaning cycles. Consequently, the durability and long-term effectiveness of lignin-based flame-retardant coatings on PET textiles remain insufficiently explored. This limitation is particularly relevant for practical applications, as coatings are susceptible to leaching and mechanical removal during repeated laundering.^25^ Moreover, to the best of our knowledge, the application of lignin-based flame-retardant systems to rPET textile substrates has not yet been reported.

In this context, the present study aims to address the flame-retardant limitations of rPET textiles through a flame-retardant coating system based on a phosphorus-based additive combined with biobased lignin and sericin (SC). Sericin, extracted from silk cocoons, was used as a nitrogen-containing synergist for the phosphorus-based flame retardant, Exolit OP 1400 (P-FR). Poly­(vinyl alcohol) (PVA) served as a water-soluble binder, while citric acid (CA) was used as a chemical cross-linker. In addition, wash durability was systematically investigated, since maintaining flame-retardant performance after repeated laundering represents a major challenge for surface-treated PET textiles and has been only rarely addressed in previous studies. Notably, the proposed system enables effective flame-retardant performance at low lignin loading while maintaining self-extinguishing behavior after repeated laundering. This approach highlights a practical pathway for developing durable and sustainable flame-retardant finishes for rPET textiles.

## 2 Experimental Section

### 2.1 Materials and Chemicals

Recycled poly­(ethylene terephthalate) plain woven fabric with an areal density of 290 g/m^2^ (abbreviated as rPET) was purchased from ZS Fabrics (Delhi, India). Poly­(vinyl alcohol) (Mowiol, degree of hydrolysis 98.0–98.8 mol %, molecular weight 195,000) abbreviated as PVA, was supplied by Sigma-Aldrich. Sericin with a crude protein content of 90% (abbreviated as SC) was obtained from Kaewluang Co., Ltd. Citric acid (purity 99.5%), abbreviated as CA, was supplied by Sigma-Aldrich. BYK-P104S, a commercial dispersant consisting of an unsaturated poly­(carboxylic acid) polymer and a polysiloxane copolymer, was obtained from BYK-Chemie GmbH. Tween 60 surfactant was purchased from Acros Organics. An aluminum diethylphosphinate-based flame retardant (Exolit OP 1400, abbreviated as P-FR) was purchased from Clariant. Alkali lignin with an average molecular weight of ∼ 10,000, sulfur content of 4%, and pH of 10.5 was obtained from Sigma-Aldrich.

### 2.2 Flame-Retardant Finishing of rPET

The flame-retardant coating solutions were prepared by dispersing the coating components listed in Table 1 in deionized water. The resulting mixtures were sonicated using an ultrasonic processor (Elmasonic Easy, Elma Schmidbauer GmbH, Germany) at 4000 rpm and 750 W for 15 min to ensure homogeneous dispersion. Prior to flame-retardant finishing, the rPET fabrics were subjected to alkaline hydrolysis using 1 M NaOH at 90 °C for 30 min. Subsequently, 25 mL of the flame-retardant dispersion was applied to each fabric sample (A4 size, 210 mm × 297 mm) using a knife-coating technique. The lignin loading in the coating formulations was expressed as % owf (on weight of fabric). The coated samples were initially dried at 90 °C for 5 min using a laboratory dryer (Model M-3, MSI Mingscape International Co., Ltd., Taiwan), after which they were turned over and dried under identical conditions on the opposite side. Finally, the samples were further dried at 120 °C for 20 min to complete the finishing process.

**Table 1:** Composition of Flame-Retardant Coating Formulations

sample	PVA (% w/v)	SC (% w/v)	CA (% w/v)	dispersant (% w/v)	surfactant (% w/v)	P-FR (% w/v)	lignin (%owf)
rPET							
L0	8.75	1	2	2.4	0.2	10	
L1	8.75	1	2	2.4	0.2	10	1
L3	8.75	1	2	2.4	0.2	10	3
L5	8.75	1	2	2.4	0.2	10	5

### 2.3 Fourier Transform Infrared (FTIR) Spectroscopy

FTIR spectra of the fabrics were collected using a Nicolet spectrometer (Thermo Scientific, USA) over a wavenumber range of 400–4000 cm^–1^. Each spectrum was acquired with 64 scans at a resolution of 4 cm^–1^. Measurements were performed in attenuated total reflectance (ATR) mode using a diamond crystal.

### 2.4 Morphological Examination

The surface morphology of the fabric samples was examined using a Schottky field-emission scanning electron microscope (FE-SEM, Hitachi SU5000) operated at an accelerating voltage of 15.0 kV. Prior to observation, the samples were sputter-coated with gold at a current of 15 mA for 60 s. Elemental composition was analyzed using the integrated energy-dispersive X-ray spectroscopy (EDS) system.

### 2.5 Thermogravimetric Analysis (TGA)

Thermogravimetric analysis was conducted to evaluate the thermal stability of the fabric samples using a thermogravimetric analyzer (TGA/DSC 2, Mettler Toledo, Switzerland). Approximately 4.3 mg of each fabric sample was heated from 25 to 800 °C at a heating rate of 10 °C/min under an air atmosphere, with an airflow rate of 50 mL/min.

### 2.6 Underwriters Laboratories 94 Vertical Burning Test (UL 94 VBT)

The flame-retardant performance of the fabric samples was evaluated according to UL 94 VBT. Five fabric specimens with dimensions of 18.5 cm × 5 cm were conditioned at 23 ± 2 °C and a relative humidity of 50 ± 10% for 48 h before testing. Each specimen was mounted vertically above a propane flame, and the flame was applied to its lower end for 10 s. The afterflame time following the first ignition (T_1_) was recorded, after which the specimen was allowed to cool for 10 s. The specimen was then exposed to the flame for an additional 10 s, and the afterflame time following the second ignition (T_2_) was recorded. During testing, observations were made regarding the occurrence of melt dripping, ignition of the cotton indicator by dripping material, and whether flaming or glowing combustion propagated upward to the holding clamp located 125 mm above the flame application point. The flame-retardant performance of the fabrics was classified based on the criteria listed in Table 2.

**Table 2:** Performance Criteria for UL 94 VBT Classifications

test criteria	V-0	V-1	V-2
afterflame time for each specimen (T_1_ or T_2_)	≤10 s	≤30 s	≤30 s
combined afterflame time for five specimens (ΣT_1_ or ΣT_2_)	≤50 s	≤250 s	≤250 s
afterflame time plus afterglow time after the second ignition for each specimen (T_2_ + T_3_)	≤30 s	≤60 s	≤60 s
flame or glowing combustion reaching the 125 mm clamping point	not allowed	not allowed	not allowed
ignition of the cotton indicator caused by flaming drips	not allowed	not allowed	not allowed

### 2.7 Limiting Oxygen Index (LOI) Test

The limiting oxygen index (LOI) was determined in accordance with ASTM D2863–06A. Rectangular fabric specimens measuring 5 cm × 10 cm were mounted vertically in a support frame and ignited at the top edge. The LOI value was defined as the minimum oxygen concentration in a flowing atmosphere required to sustain continuous sample burning. Average LOI values were calculated from three independent measurements.

### 2.8 Durability against Washing

The durability of flame-retardant performance against washing was evaluated according to AATCC 61/2A. Each fabric sample was sewn to a multifiber adjacent fabric and washed using 0.15% detergent (AATCC 1993 Standard Reference Detergent WOB) in 150 mL of water containing 50 steel balls at 49 °C for 45 min. In addition to flame-retardancy evaluation, color fastness was assessed using the gray scale for color change and the gray scale for color staining.

### 2.9 Durability against Crocking

The durability of the flame-retardant coating against crocking was evaluated by conducting dry and wet crocking tests using a crockmeter (James H. Heal & Co., Ltd.) in accordance with AATCC TM8–2016e. For the dry crocking test, a dry cotton cloth was mounted on the rubbing finger of the crockmeter and rubbed against the fabric sample for 10 cycles under an applied load of 9 ± 1 N. For the wet crocking test, the same procedure was followed, except that the cotton rubbing cloth was wetted to achieve a moisture content of 65 ± 5% before testing. The stained cotton cloths were subsequently evaluated using the gray scale for color staining.

### 2.10 Contact Angle Measurement

Static water contact angles (WCA) on the fabric samples were measured using a tensiometer (Attension, Finland) with a water droplet volume of 3 ± 0.5 μL. Contact angles were recorded 1 s after droplet deposition to ensure consistent comparison among samples. The reported WCA values were obtained by averaging the left and right contact angles of the sessile droplet.

### 2.11 Tensile Properties

The tensile strength and elongation at break of the fabric samples were measured in accordance with ASTM D5035–11 (2019). Fabric specimens with dimensions of 5 cm × 15 cm were subjected to tensile testing using a universal testing machine (Zwick/Roell Z005, Germany) with a grip separation of 75 mm, a crosshead speed of 300 mm/min, and a 5 kN load cell. Each reported value represented the average of five replicate measurements.

## 3 Results and Discussion

### 3.1 Morphological Observation

Figure 1 illustrates the surface morphology of rPET fabrics before and after coating with flame-retardant dispersions containing lignin. As shown in Figure 1b, the woven structure of the rPET fabric was almost completely covered by the applied coating layer.

**Figure 1:**
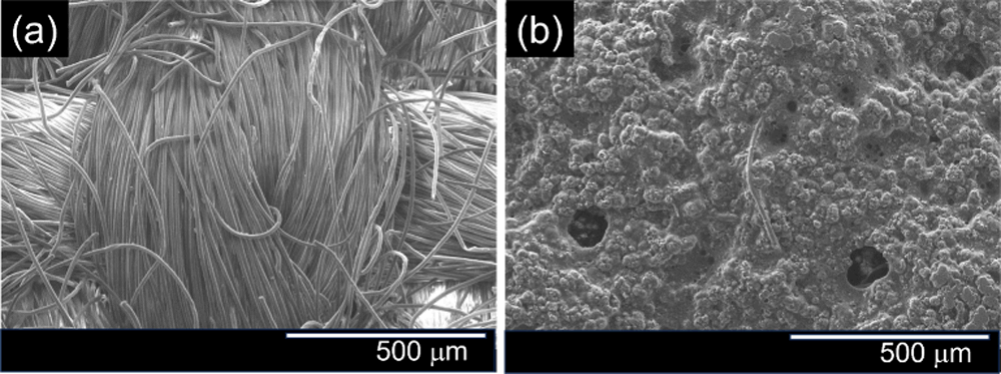
SEM images of (a) untreated fabric and (b) L3.

Figure 2 presents the EDS elemental mapping image of L3, confirming the presence of phosphorus (P) and aluminum (Al), which are the principal elements of the phosphorus-based flame-retardant additive (P-FR). Both P and Al were uniformly distributed across the fabric surface, indicating homogeneous deposition of the flame-retardant coating. The corresponding elemental weight percentages are summarized in Table 3. The detected phosphorus content was higher than that of aluminum and sulfur, respectively. The P and Al signals originate primarily from P-FR, whereas the sulfur signal is mainly attributed to the sulfur-containing alkali lignin used in the formulation (≈4 wt % S according to the supplier specification), with a possible minor contribution from SC. Although SEM–EDS is a semiquantitative technique that detects only elements near the fiber surface,^26^ a phosphorus content exceeding 2 wt % is sufficient to impart significant flame-retardant properties to the treated fabrics.^27^

**Figure 2:**
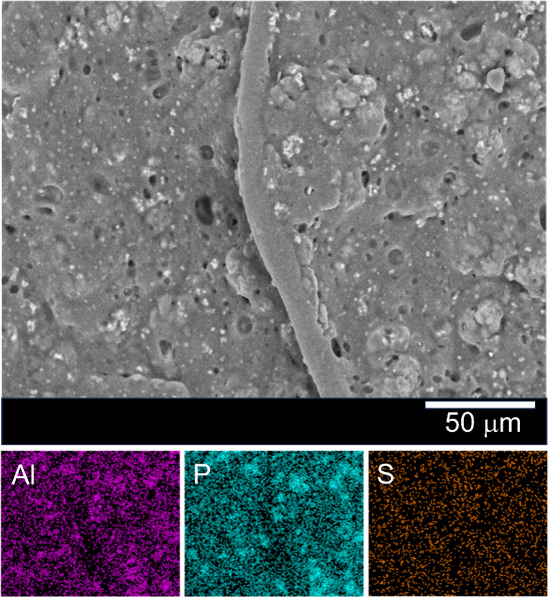
EDS elemental mapping of L3, showing the distribution of Al, P, and S.

**Table 3:** Elemental Composition of Coated Fabrics Determined by SEM-EDS

sample	elemental composition (wt %)				
	C	O	P	Al	S
L3	62.0	34	2.6	1.1	0.2

### 3.2 FTIR Spectra

FTIR spectra of the individual components in the flame-retardant coatings are presented in Figure 3a, while the spectra of untreated and treated rPET fabrics are shown in Figure 3b. The spectra of PVA, CA, and lignin exhibit O–H stretching vibrations at 3200–3500 cm^–1^, with varying intensities.^28^ Weak absorption bands attributed to C–H stretching vibrations were observed at 2909–2967 cm^–1^, which are characteristic of organic compounds.^29^ The P-FR exhibited characteristic absorption bands at 1179 and 1021 cm^–1^, which can be attributed to P=O and P–O stretching vibrations, respectively.^30^ SC exhibited a strong absorption peak at 1620 cm^–1^ corresponding to the amide I (C=O stretching) vibration, together with characteristic amide bands at 1530 cm^–1^ (amide II) and 1390 cm^–1^ (amide III).^31^ In Figure 3b, the FTIR spectrum of untreated rPET shows a prominent carbonyl stretching peak at 1710 cm^–1^ and a C–O–C stretching vibration at 1240 cm^–1^, which is characteristic of polyester materials.^29^ The appearance of absorption bands corresponding to O–H, C=O, P=O, and Al–O stretching vibrations in the treated fabrics confirms the successful deposition of the flame-retardant coating.

**Figure 3:**
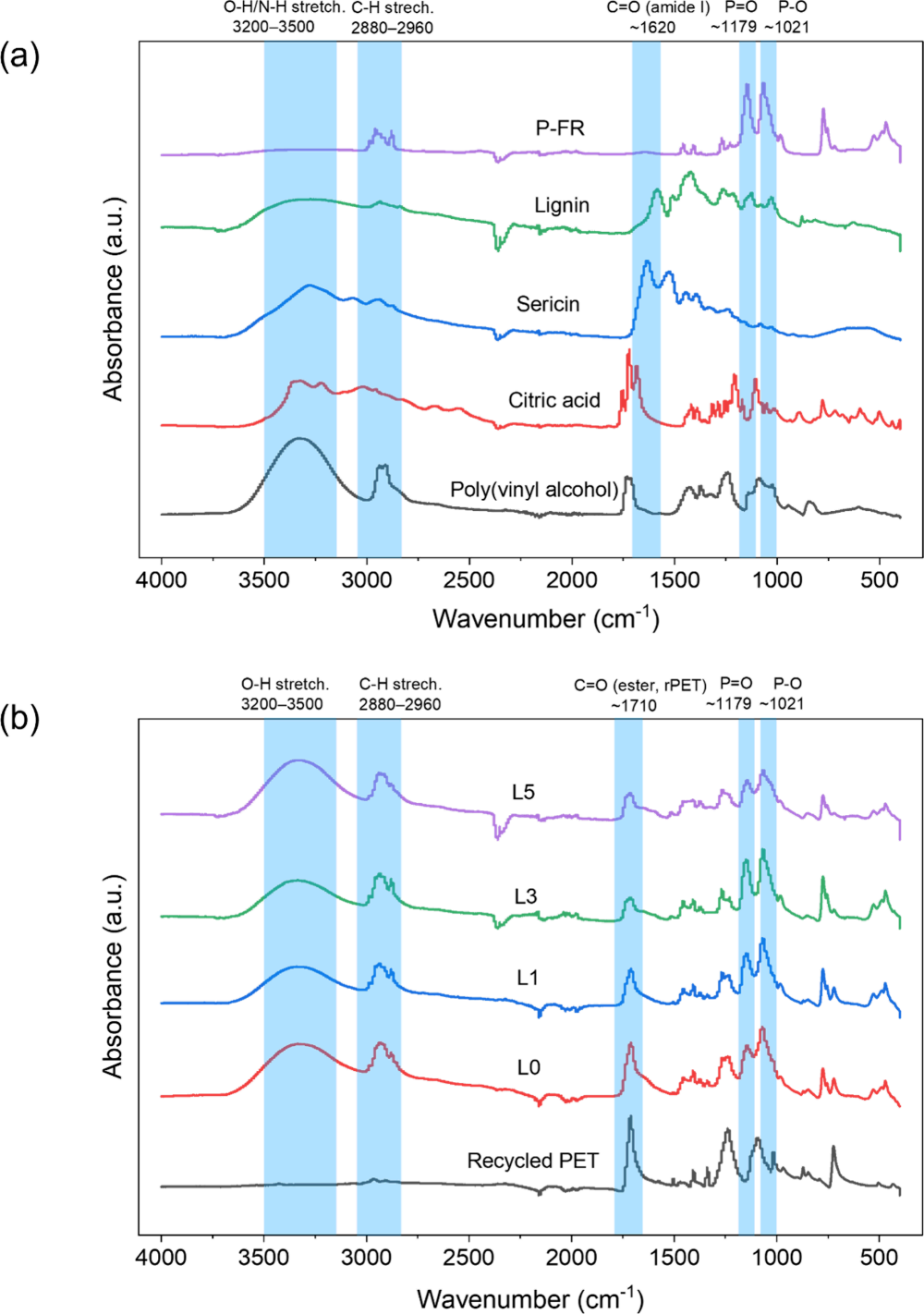
ATR-FTIR spectra of (a) the individual flame-retardant components and (b) untreated and treated fabric samples. L0–L5 denote the lignin loading used in the coating formulation.

### 3.3 Flame-Retardant Performance and Thermal Behavior

#### 3.3.1 Thermogravimetric Analysis (TGA)

TGA was performed to evaluate the thermal stability and oxidative degradation behavior of untreated and treated rPET fabrics under an air atmosphere. The corresponding TGA and derivative thermogravimetric (DTG) curves are presented in Figure 4a,b, respectively. Key thermal parameters extracted from the TGA data are summarized in Table 4, including the lignin-related decomposition peak temperature (T_pL_), the primary and secondary degradation peak temperatures (T_p1_ and T_p2_), and the char residue at 800 °C. According to the TGA curves shown in Figure 4a, untreated rPET exhibited two distinct degradation stages, with the first peak (T_p1_) at approximately 436.5 °C, attributed to polymer chain scission, and the second peak (T_p2_) at 597.67 °C, associated with oxidative degradation of the residual char.^32^ Both T_p1_ and T_p2_ shifted to lower temperatures for the treated samples, which can be attributed to the generation of phosphorus-containing acidic species from P-FR, which catalyzed the dehydration and carbonization of the fibers. This catalytic process promoted the formation of a phosphorus-rich char layer on the rPET surface. Therefore, the reduced T_p_ values observed for the treated samples did not indicate inferior flame-retardant performance. Samples treated with higher lignin contents of 3–5% owf exhibited an additional minor DTG peak (T_p,L_) near 300 °C, which is attributable to lignin-related decomposition within the coating layer.^33^

**Table 4:** TGA and DTG Results of Untreated and Treated rPET Fabrics

sample	T_p,L_	T_p1_	T_p2_	residual mass at 800 °C (%)
rPET		436.5	597.7	0.95
L0		427.2	548.5	8.25
L1		427.5	564.2	6.23
L3	304.3	431.5	535.8	8.32
L5	300.5	426.5	526.0	10.15

**Figure 4:**
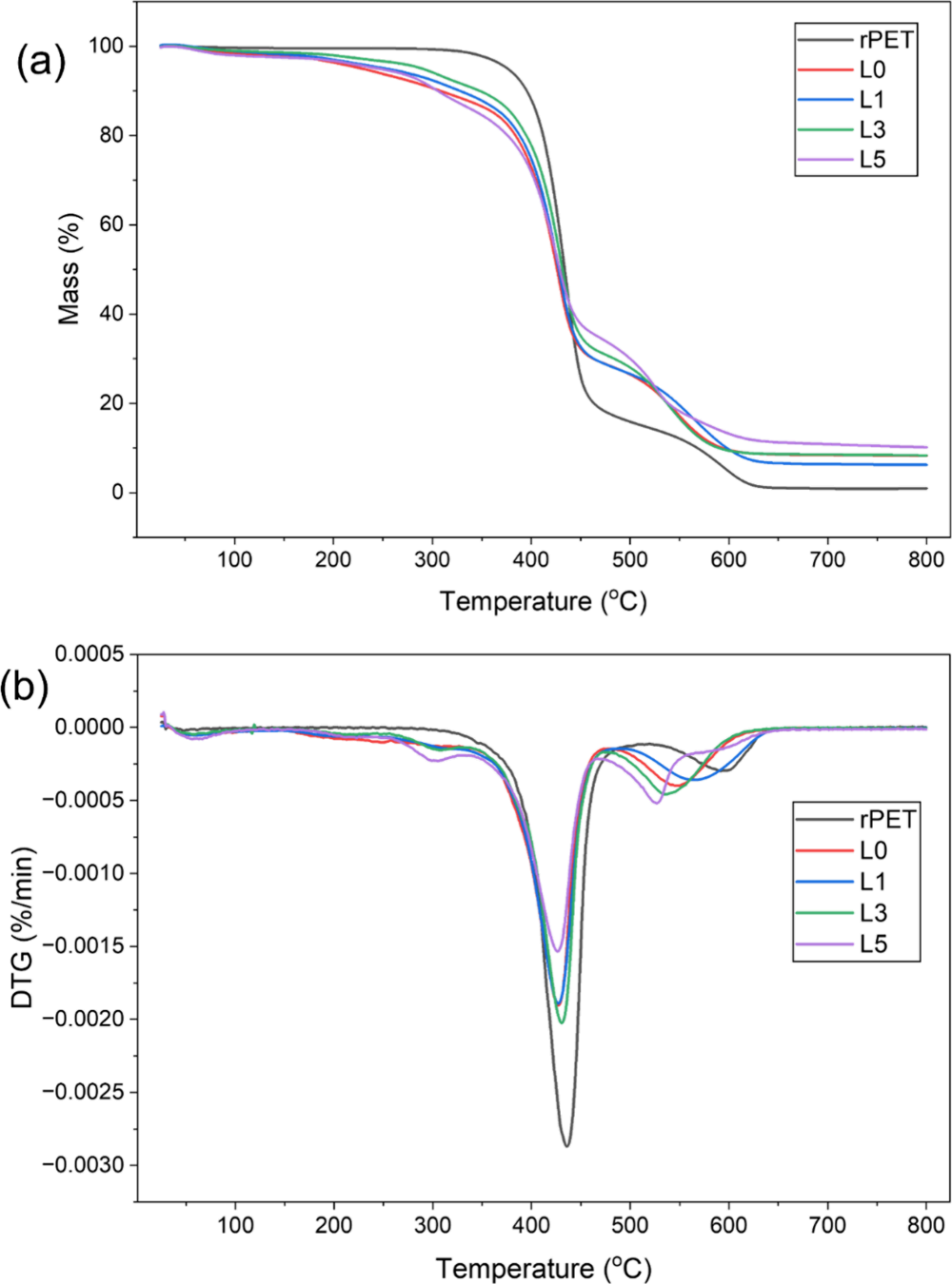
Thermogravimetric analysis of untreated and treated rPET fabrics under an air atmosphere: (a) TGA curves and (b) corresponding DTG curves. L0–L5 denote the lignin loading used in the coating formulation.

Higher char residues indicate enhanced flame resistance due to the formation of a protective carbonaceous layer that limits heat and oxygen transfer. As shown in Table 4, untreated rPET produced a negligible char residue at 800 °C (0.95%), which is expected because PET primarily decomposes into volatile products.^34^ These volatile degradation products contribute to rapid flame propagation and may pose inhalation hazards. In contrast, the sample treated with the flame-retardant formulation without lignin (L0) exhibited a significantly higher char residue of 8.25%. This enhancement is attributed to the presence of SC, CA, PVA, and most importantly P-FR, which collectively served as carbon sources and promoted char formation. SC also functioned as a nitrogen source, facilitating the release of nonflammable gases, while P-FR played a dominant role in increasing char residue through a condensed-phase flame-retardant mechanism. Furthermore, the incorporation of lignin led to a further increase in char residue. As summarized in Table 4, the residual mass increased from 8.25% for L0 to 10.15% for L5. This behavior is associated with the highly aromatic structure of lignin, which favors the formation of thermally stable carbonaceous char layers that act as a protective barrier, thereby shielding the underlying rPET fibers from heat and oxygen.

#### 3.3.2 UL 94 Vertical Burning Test

Table 5 summarizes the UL 94 VBT results for untreated rPET fabrics and fabrics treated with flame-retardant coatings. The untreated rPET fabric could not be classified according to UL 94 criteria, as flame propagation reached the holding clamp, and pronounced melt-dripping behavior was observed. When the fabrics were coated with a P-FR-containing formulation without lignin (L0), a V-2 rating was achieved, indicating self-extinguishing behavior accompanied by molten drips that ignited the cotton indicator positioned below the specimen. This sample exhibited the highest total afterflame time of 69.8 s among all treated fabrics. The introduction of lignin into the coating formulation improved flame retardancy to a V-0 rating, demonstrating complete self-extinguishing behavior without observable melt dripping. It should be noted that the addition of only 1% owf lignin (L1) reduced the total afterflame time to 12.8 s, which represented the lowest value recorded in this study. However, higher lignin loadings of 3–5% owf did not further reduce the total afterflame time and, in some cases, resulted in inferior performance. These inconsistencies were attributed to nonuniform coating formation, as increased lignin content likely produced thicker yet more fragile char layers. During combustion, such char layers may partially crack or detach from the fiber surface, thereby allowing flame penetration to the underlying material. Representative photographic images of the tested fabric samples are presented in Table 6.

**Table 5:** UL 94 Vertical Burning Test Results of rPET Fabrics

sample	ΣT1 + ΣT2 (s)	UL 94 rating
rPET		not rated
L0	69.8	V-2
L1	12.8	V-0
L3	32.1	V-0
L5	30.2	V-0

**Table 6:**
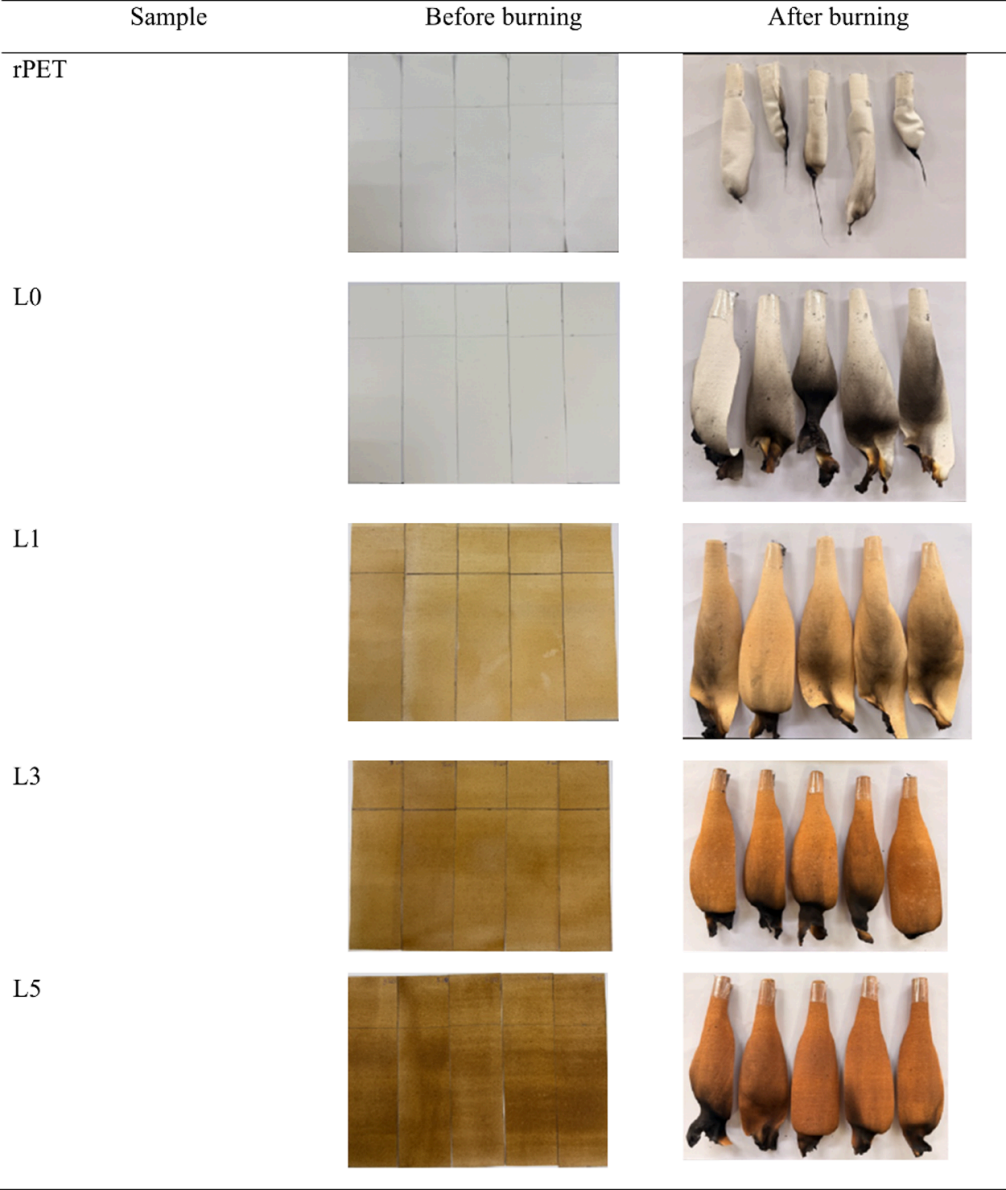
Photographic Images of Samples before and after UL 94 VBT Testing

No afterglow was observed for any of the coated fabrics (T3 = 0 s), indicating that combustion ceased immediately after flame removal. Therefore, the UL 94 rating was determined solely by the measured afterflame times (T1 and T2).

#### 3.3.3 Limiting Oxygen Index (LOI)

LOI values were measured to quantitatively evaluate the flame retardancy of PET fabrics after flame-retardant finishing. Higher LOI values indicate a greater oxygen concentration required to sustain combustion, reflecting improved flame-retardant performance. As shown in Table 7, the untreated rPET fabric exhibited the lowest LOI value of 26.0%, which is insufficient to achieve self-extinguishing behavior under ambient conditions.^35^ For the fabric treated with the flame-retardant formulation without lignin (L0), the LOI value increased to 31.1%, indicating self-extinguishing behavior in normal air. This improvement is attributed to the presence of aluminum diethylphosphinate in P-FR, which promoted the formation of a phosphorus- and aluminum-rich char layer. Such a char layer reduced heat and oxygen transfer, thereby increasing the oxygen concentration required to sustain combustion. For samples treated with lignin loadings ranging from 1–5% owf, the LOI values increased from 37.5 ± 0.1% to 47.0 ± 0.0%. This enhancement is attributed to the highly aromatic and carbon-rich structure of lignin, which facilitates the formation of a stable carbonaceous residue. The resulting char layer further increased the oxygen concentration required to sustain burning, thereby significantly improving flame retardancy.^36^ Statistical analysis (one-way ANOVA followed by Tukey’s HSD test, *p* < 0.05) confirmed that the LOI values of all treated samples were significantly higher than those of untreated rPET.

**Table 7:** Limiting Oxygen Index (LOI) Values of Untreated and Treated PET Fabrics[Table-fn t7fn2]

sample	LOI (%O_2_)
rPET	26.0 ± 0.0^a^
L0	31.1 ± 0.0^b^
L1	37.5 ± 0.1^c^
L3	39.4 ± 0.0^d^
L5	47.0 ± 0.0^e^

a Values are expressed as mean ± standard deviation (*n* = 3). Different superscript letters indicate significant differences according to Tukey’s HSD test at *p* < 0.05.

#### 3.3.4 Morphological Analysis of Char Residues

Figure 5 presents SEM images of the char residues which provide additional insight into the flame-retardant mechanism of the coated rPET fabrics. The untreated rPET sample exhibits fused molten structures typical of thermoplastic polymers undergoing melt dripping during combustion, while the binder-treated fabric (PVA/CA/SC) shows a relatively dense molten morphology without a well-developed porous char structure. In contrast, the incorporation of the phosphorus-based flame retardant generates a porous carbonaceous structure with bubble-like features, indicating a condensed-phase flame-retardant mechanism. When lignin is introduced into the coating formulation, the char morphology becomes more compact with finer porous features (L1), suggesting that lignin contributes additional carbon to the char matrix. At higher lignin loading (L5), the char residue appears thicker but more heterogeneous with larger pores, indicating a less compact protective layer. Such differences in char morphology help explain the UL 94 VBT results, where higher lignin loadings (3–5% owf) did not further reduce the afterflame time despite higher LOI values. The coarser char structure observed for L5 may reduce the barrier effectiveness under direct flame exposure, allowing heat and oxygen to reach the underlying substrate and thereby prolonging the afterflame time. These results suggest that the synergistic action of phosphorus-based species and lignin-derived carbon promotes char formation, while an optimal lignin content (L1) provides the most effective protective barrier.

**Figure 5:**
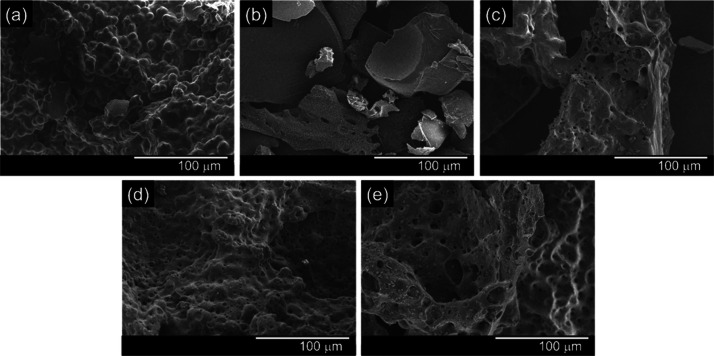
SEM images of the combustion residues of (a) rPET, (b) binder-treated fabric (PVA/CA/SC), (c) L0, (d) L1, and (e) L5.

The elemental compositions of the combustion residues determined by SEM-EDS are summarized in Table 8. The chars of untreated rPET and the binder-treated fabric consisted mainly of carbon and oxygen, reflecting the decomposition of the polyester matrix and organic binders. In contrast, L0, L1, and L5 exhibited detectable phosphorus and aluminum signals originating from P-FR, indicating that the phosphorus-based flame retardant remained in the condensed phase and contributed to char formation during combustion. This observation is consistent with the elemental mapping results shown in Figure 2, which confirmed the presence and uniform distribution of Al and P in the coated fabrics prior to combustion. A small sulfur signal was detected in L5, which is attributed to the sulfur-containing impurities in lignin. The slightly lower phosphorus and aluminum contents in L5 compared with L0 and L1 are likely due to dilution by the higher lignin content in the coating formulation.

**Table 8:** Elemental Composition (wt%) of Combustion Residues Determined by SEM-EDS[Table-fn t8fn2]

sample	elemental composition (wt %)				
	C	O	P	Al	S
rPET	67.7	32.3	0.0	0.0	0.0
binder-treated fabric	74.9	25.0	0.0	0.0	0.0
L0	65.6	24.1	6.1	4.2	0.0
L1	64.2	25.6	5.7	4.4	0.0
L5	69.0	24.2	4.1	2.4	0.2

a Minor trace elements (<0.2 wt %) were omitted for clarity.

### 3.4 Wash Durability

Table 9 presents the flame-retardant performance of the treated fabrics after five equivalent washing cycles. Flame retardancy was evaluated based on total afterflame time and UL 94 VBT classification. All washed samples exhibited increased total afterflame times, indicating partial loss of the flame-retardant coating during laundering. After washing, all fabrics were classified as V-2, demonstrating self-extinguishing behavior accompanied by melt dripping.

**Table 9:**
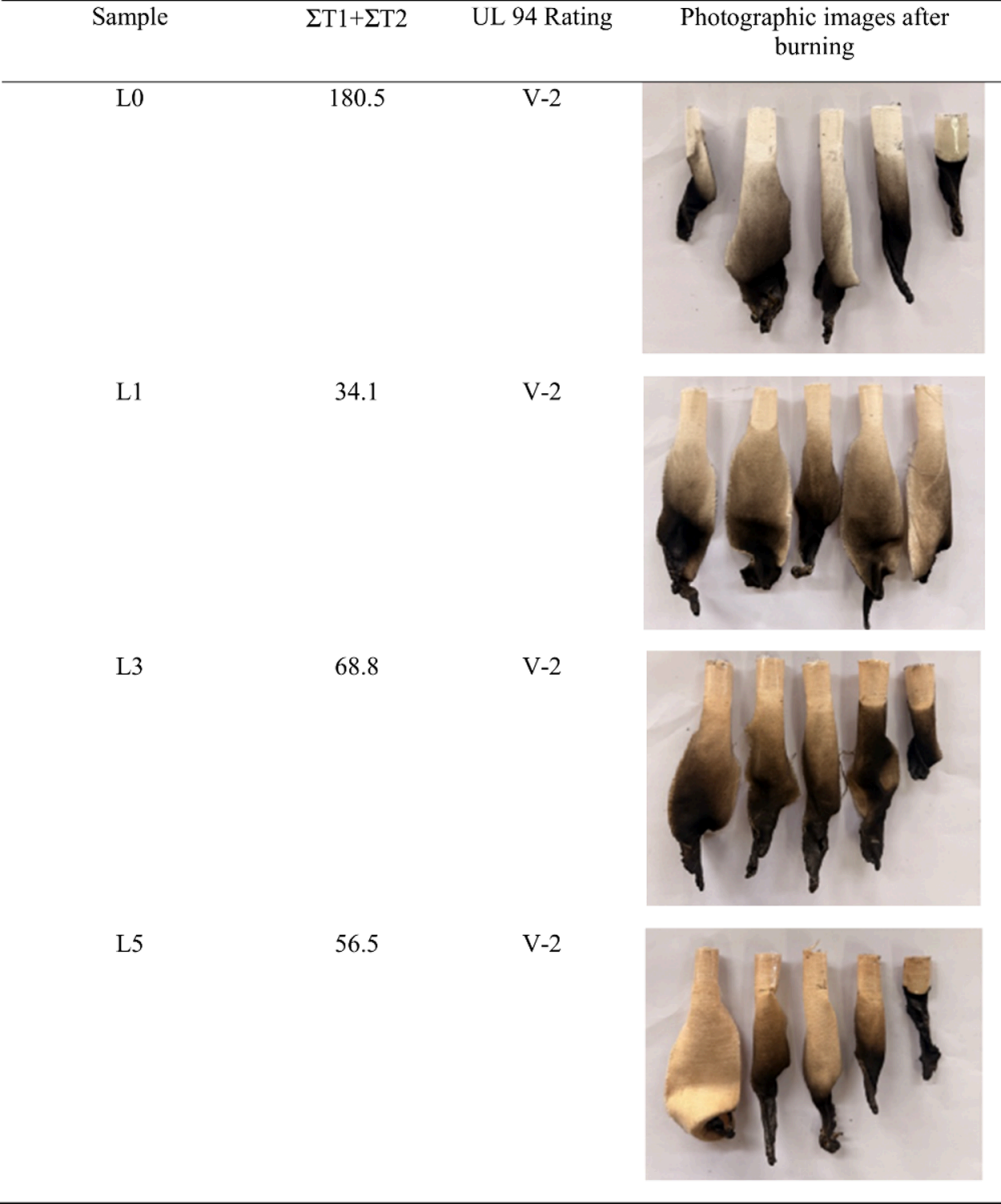
Afterflame Time and UL 94 VBT Classification of PET Fabrics after Five Laundering Cycles

Lignin played a key role in reducing afterflame time following washing. The fabric treated without lignin exhibited the highest total afterflame time of 180.5 s, whereas the incorporation of only 1% owf lignin reduced the afterflame time to 34.1 s. Further increases in lignin loading to 3–5% owf did not result in additional reductions in afterflame time, in contrast to the LOI results, where higher lignin contents led to progressively higher LOI values. This discrepancy can be attributed to the fundamentally different flame-retardant behaviors evaluated by the two test methods. While LOI primarily reflects the amount of char formed and the oxygen concentration required to sustain combustion, the UL 94 VBT is highly sensitive to the integrity and mechanical stability of the char layer under rapid flame exposure. Higher lignin loadings likely produced thicker but more fragile char layers, which were more susceptible to cracking or partial detachment during burning. This degradation allowed heat and oxygen to reach the underlying substrate, thereby prolonging afterflame time. Based on UL 94 VBT performance and wash durability, the L1 formulation was identified as the optimal system.

### 3.5 Color Fastness Properties

Fabric samples were evaluated for color fastness to washing, and the corresponding color fastness ratings are reported in Table 10. As lignin was the sole colorant in the coating system, imparting a light brown coloration, increasing lignin content was expected to adversely affect both color change and color staining ratings.

**Table 10:** Color Fastness to Washing Ratings According to AATCC 61/2A

		color staining					
sample	color change	wool	acrylic	PET	nylon	cotton	acetate
L0	5	5	5	5	5	5	5
L1	2	4–5	4–5	5	3–4	4–5	4–5
L3	2	3–4	4	3–4	2–3	4	3–4
L5	1	3	2–3	3	2	3–4	3–4

The degree of fabric fading was assessed by comparing the washed samples with the gray scale for color change. The gray scale ratings decreased from poor (2) to very poor (1) as the lignin content increased from 1% owf to 5% owf.

Color staining of the adjacent multifiber fabric was evaluated by comparing the grayscale with the color staining. The staining tendency varied among different fiber components within the multifiber fabric. Nylon exhibited the highest susceptibility to staining, which can be attributed to its amino functional groups and high absorbance. Lignin molecules were likely adsorbed onto nylon fibers through hydrogen bonding between the amino group of nylon and the phenolic groups of lignin. Overall, the observed trends in color change ratings were consistent with the color staining results, confirming that increasing lignin content led to decreased color fastness to washing.

The partial loss of lignin during washing is more likely associated with the swelling of the PVA-based coating matrix in water. Although PVA was cross-linked with CA, the resulting network remains hydrophilic and can undergo limited swelling during laundering. As a result, weakly bound lignin particles embedded in the coating layer may be partially released, leading to fabric fading and potential staining of adjacent multifiber fabrics.

Color fastness to crocking results are summarized in Table 11. The treated fabrics were subjected to rubbing using a cotton crocking cloth, and the degree of color staining on the cloth was evaluated using the gray scale for color staining, where ratings range from 1 (very poor) to 5 (very good). Higher ratings indicate better color fastness to crocking. In this system, color transfer was attributed to the presence of lignin in the coating. All samples achieved a rating of 5 under dry crocking conditions, as no visible color staining was detected on the crocking cloth. However, under wet crocking conditions, samples containing lignin exhibited lower ratings, although all values remained above 3, indicating acceptable color fastness. The reduced ratings under wet conditions are attributed to enhanced transfer of water-soluble lignin from the fabric surface to the crocking cloth in the presence of moisture.

**Table 11:** Color Fastness to Crocking Ratings According to AATCC TM8-2016e

	color fastness to crocking (gray scale)	
sample	dry	wet
L0	5	5
L1	5	3–4
L3	5	3
L5	5	4–5

### 3.6 Water Contact Angle Measurement

Table 12 summarizes the water contact angle (WCA) values of untreated and treated fabrics. The alkaline-treated rPET fabric without coating absorbed the water droplet immediately due to strong capillary effects within the fibrous structure. In contrast, the sample treated with the flame-retardant formulation without lignin (L0) exhibited a high WCA of 143.84°, indicating a highly hydrophobic surface. Although the coating formulations contained hydrophilic components such as SC, PVA, and CA, the applied coating reduced capillary absorption by forming a coating layer that partially covers the fabric surface (Figure 1b) and may reduce capillary penetration through interfiber pathways.^37^ With increasing lignin loading from 0 to 5% owf, the WCA values gradually decreased. This trend is attributed to the intrinsic polarity of lignin, which contains abundant phenolic and aliphatic hydroxyl groups. The incorporation of lignin therefore increased the surface polarity of the coating layer, leading to enhanced surface wettability and a gradual reduction in WCA. Nevertheless, all treated fabrics remained hydrophobic, with WCA values exceeding 90°, indicating that the coating maintained adequate water repellency for practical applications.

**Table 12:**
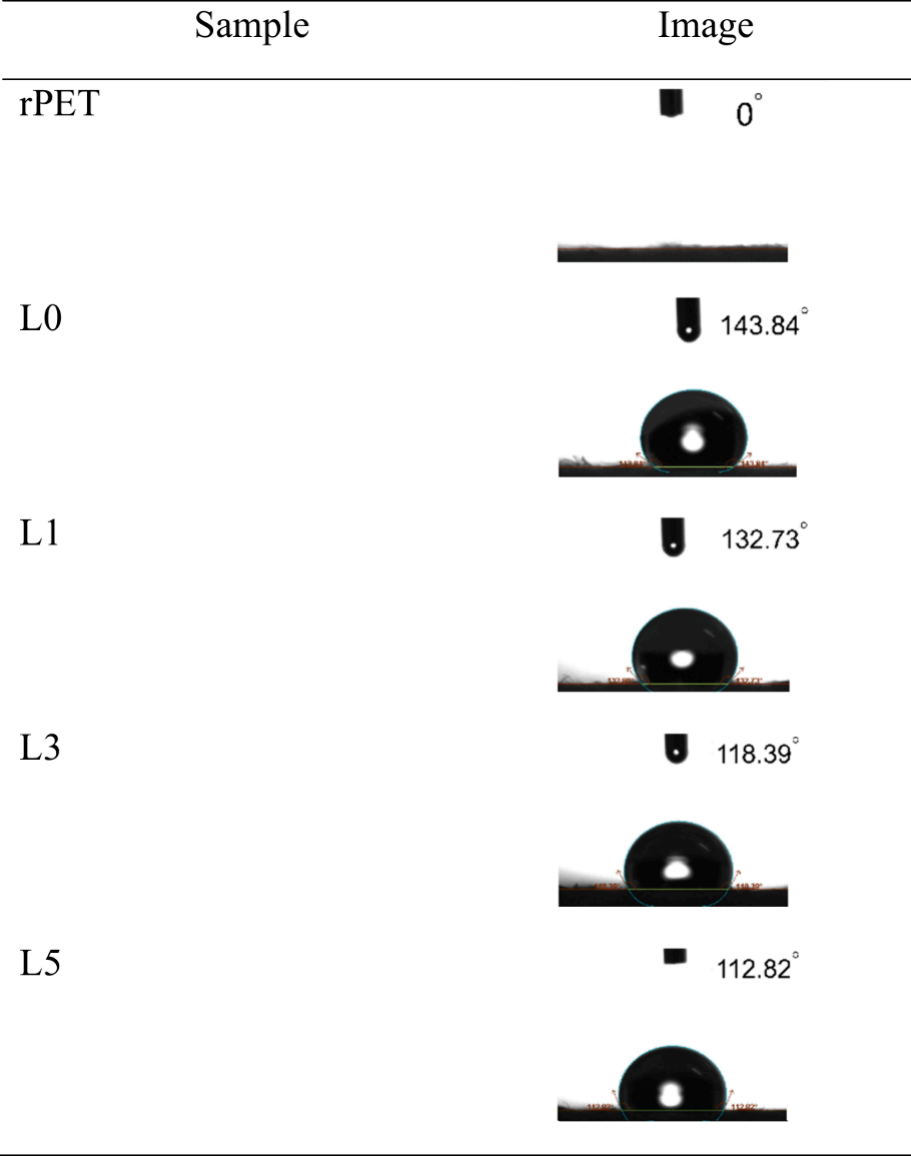
Water Contact Angle (WCA) of Untreated and Treated rPET Fabrics

### 3.7 Mechanical Properties

Table 13 presents the tensile properties of untreated and treated rPET fabrics. The sample treated without lignin (L0) exhibited tensile strength and elongation at break comparable to those of untreated rPET, indicating minimal influence of the binder on fiber structure and yarn mobility. In contrast, lignin-containing coatings led to a gradual increase in tensile strength in the weft direction with increasing lignin content, with L3 and L5 showing significantly higher values than untreated rPET (*p* < 0.05), while L0 and L1 showed no significant differences.

**Table 13:** Tensile Properties of rPET Fabrics Measured in Accordance with ASTM D503511 (2019)[Table-fn t13fn2]

sample	warp direction		weft direction	
	tensile strength (N)	elongation (%)	tensile strength (N)	elongation (%)
rPET	2683 ± 26^a^	39.9 ± 1.6^a^	1177 ± 78^a^	27.4 ± 0.9^a^
L0	2733 ± 215^a^	31.9 ± 3.2^b^	1188 ± 112^ab^	29.1 ± 3.0^a^
L1	2370 ± 147^b^	37.7 ± 1.1^a^	1260 ± 85^ab^	20.2 ± 1.1^b^
L3	2276 ± 114^b^	38.9 ± 2.6^a^	1400 ± 112^b^	19.7 ± 1.6^b^
L5	2277 ± 92^b^	41.3 ± 3.2^a^	1421 ± 162^b^	22.2 ± 2.8^b^

a Values are expressed as mean ± standard deviation (*n* = 5). Different superscript letters within the same column indicate significant differences according to Tukey’s HSD test at *p* < 0.05.

In the warp direction, however, tensile strength decreased with lignin incorporation. This can be attributed to the structural characteristics of the fabric, where warp yarns, having lower crimp and higher initial tension, are less capable of redistributing stress. The increased coating rigidity therefore promotes earlier failure under tensile loading, indicating a direction-dependent mechanical response.

In addition to tensile strength, elongation at break was evaluated to assess fabric extensibility. In the warp direction, only L0 exhibited a significantly lower elongation than untreated rPET (*p* < 0.05), while lignin-containing samples remained comparable. In contrast, in the weft direction, L1–L5 showed significantly lower elongation at break despite increased tensile strength, indicating that lignin incorporation enhanced load-bearing capacity while restricting yarn mobility and extensibility.

### 3.8 Comparison with Lignin-Based Flame-Retardant Systems in PET Textiles

A comparison of lignin-based flame-retardant systems reported for PET textiles and the present study is summarized in Table 14. Overall, previously reported systems demonstrate the potential of lignin for enhancing flame retardancy in polyester-based substrates, as reflected by reductions in heat release behavior or improvements in self-extinguishing characteristics. The results obtained in this work further demonstrate that effective flame-retardant performance can be achieved on rPET fabrics using a relatively low lignin loading. The treated fabrics achieved a UL 94 V-0 classification with an LOI value of 37.5% at only 1% owf lignin, indicating efficient char formation and suppression of melt dripping. In addition, flame-retardant performance was retained after repeated laundering, confirming partial durability of the coating under practical conditions. As summarized in Table 14, only a limited number of studies have addressed this aspect, particularly with respect to performance after repeated laundering. Furthermore, the treated fabrics maintained overall mechanical performance. These results highlight the effectiveness of integrating lignin within a phosphorus–nitrogen-based system for developing sustainable flame-retardant finishes for rPET textiles.

**Table 14:** Comparison of Flame-Retardant Performance of Lignin-Based Systems Reported in the Literature and This Work (TS = Tensile Strength; EB = Elongation at Break; PHRR = Peak Heat Release Rate; NR = Not Reported; PU = Polyurethane)

substrate/coating system	flame retardant additive	LOI	flammability test	wash durability	tensile properties	reference
PET fabric/sodium lignosulfonate	sodium lignosulfonate (200 g/L)	27	cone calorimetry: 82% reduction in PHRR; UL 94 VBT: self-extinguishing without melt dripping	yes (2 dry cleaning cycles)	NR	Basak et al.^22^
PET fabric/lignin nanoparticles	lignin nanoparticles (2 g/L)	NR	microscale combustion calorimetry: PHRR reduction 25.15–37.66%	NR	TS increased 4.14–12.28% EB increased 30.24–48.48% (yarn direction not specified)	Ouadil et al.^23^
PET fabric/lignin/PU binder	APP (10 wt %) + lignin (10 wt %)	NR	cone calorimetry: 59% reduction in PHRR	NR	NR	Giraud et al.^24^
rPET fabric/alkali lignin/SC/P-FR/PVA binder	alkali lignin (1% owf) + SC (1% w/v) + P-FR (10% w/v)	37.5	UL 94 VBT: V-0 before washing; V-2 after 5 washing cycles	yes (5 washing cycles)	TS increased (weft) and decreased (warp); EB decreased (weft), retained (warp)	present study

## 4 Conclusions

This study demonstrates a sustainable and effective strategy for producing flame-retardant recycled polyester (rPET) textiles using a halogen-free coating system composed of a phosphorus-based flame retardant (P-FR), biobased lignin, sericin, and a PVA/citric acid matrix. The incorporation of lignin as a char-forming component, in combination with the phosphorus–nitrogen system, promoted char formation, suppressed melt dripping, and enabled self-extinguishing behavior. Notably, the addition of only 1% owf lignin was sufficient to achieve a UL 94 V-0 rating, with the LOI increasing from 26.0% to 37.5%, demonstrating effective flame-retardant performance at low lignin loading. Increasing lignin content to 5% owf further increased the LOI to 47.0%, although no additional improvement in UL 94 performance was observed.

After five laundering cycles, all treated fabrics retained self-extinguishing behavior, despite a reduction in UL 94 classification to V-2, indicating partial loss of the coating. The treated fabrics also retained hydrophobicity and acceptable mechanical performance, confirming that the coating system did not adversely affect the functional properties of the textile substrate.

Overall, these findings demonstrate that low lignin loading can provide effective and partially durable flame retardancy in rPET textiles, highlighting a practical and sustainable approach for textile finishing applications.

## Supplementary Material



## Notes

Raw ATR-FTIR data of flame-retardant components and untreated/treated rPET fabric samples, and raw TGA data of untreated and treated rPET fabric samples (XLSX)
